# Ring Char Formation During High‐Power, Short‐Duration Ablation Using an Irrigated Six‐Hole Contact Force Sensing Tip Catheter

**DOI:** 10.1002/joa3.70301

**Published:** 2026-02-16

**Authors:** Yasuteru Yamauchi, Yuichiro Sagawa, Kazuya Murata, Hirofumi Arai, Atsuhito Oda, Kazutaka Aonuma

**Affiliations:** ^1^ Department of Cardiology Japan Red Cross Yokohama City Bay Hospital Kanagawa Japan; ^2^ Department of Cardiology Mito Saiseikai General Hospital Ibaraki Japan

**Keywords:** ablation, atrial fibrillation, char, contact force, high‐power short‐duration

## Abstract

During circumferential ablation of the right pulmonary veins (PVs), high‐power, short‐duration (HPSD) ablation at the anterior wall of the right PVs, corresponding to the left atrial septal side, resulted in an abrupt impedance increase from 120 to 180 Ω, occurring 11 s after RF delivery initiation (A). After an abrupt impedance rise, withdrawal of the ablation catheter from the long sheath revealed a circumferential thrombus adhering directly above the irrigation holes at the catheter tip (B). The catheter tip after thrombus removal is shown in (C), and the ring‐shaped thrombus that adhered to the catheter tip is displayed in (D).

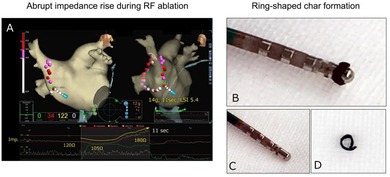

## 1 Introduction

High‐power, short‐duration (HPSD) radiofrequency (RF) ablation has become increasingly popular for pulmonary vein isolation (PVI) in atrial fibrillation (AF) because it enhances resistive heating, producing durable and localized lesions while shortening procedure time [1]. Several studies have confirmed its safety and efficacy compared with conventional ablation. However, high‐power delivery carries risks such as steam pops, impedance rise, or catheter tip charring, potentially increasing thromboembolic risk. Makimoto et al. linked charring to excessive contact force, prolonged applications, and gradual impedance rise, particularly at the anteroinferior right pulmonary vein (PV) [2].

## 2 Case Report

**FIGURE 1:**
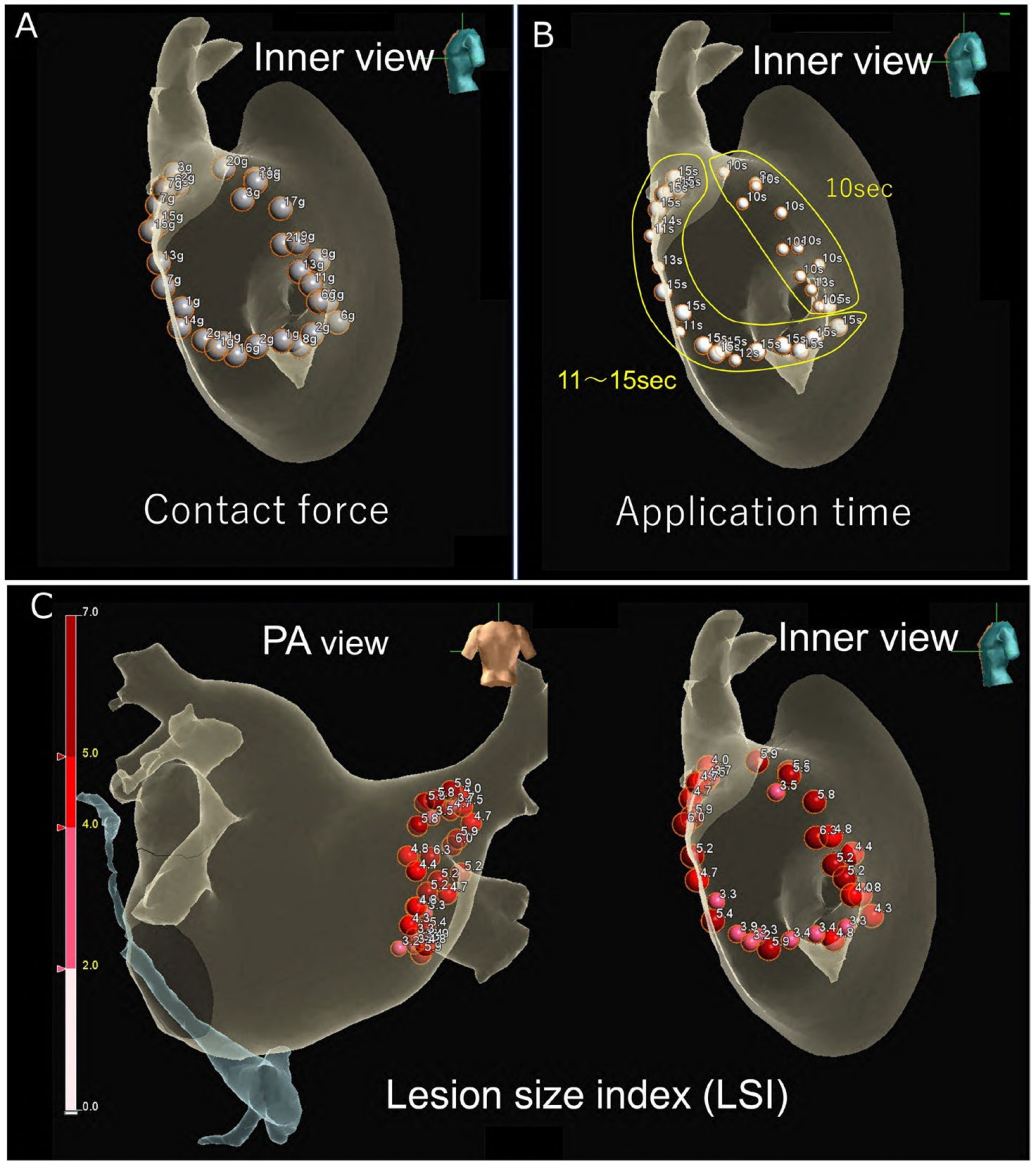
Ablation data obtained during high‐power, short‐duration ablation for right pulmonary vein isolation. (A) Contact force, (B) application time, and (C) lesion size index at each ablation site are shown on a 3D image of the EnSite system in both the inner and posteroanterior views.

**FIGURE 2:**
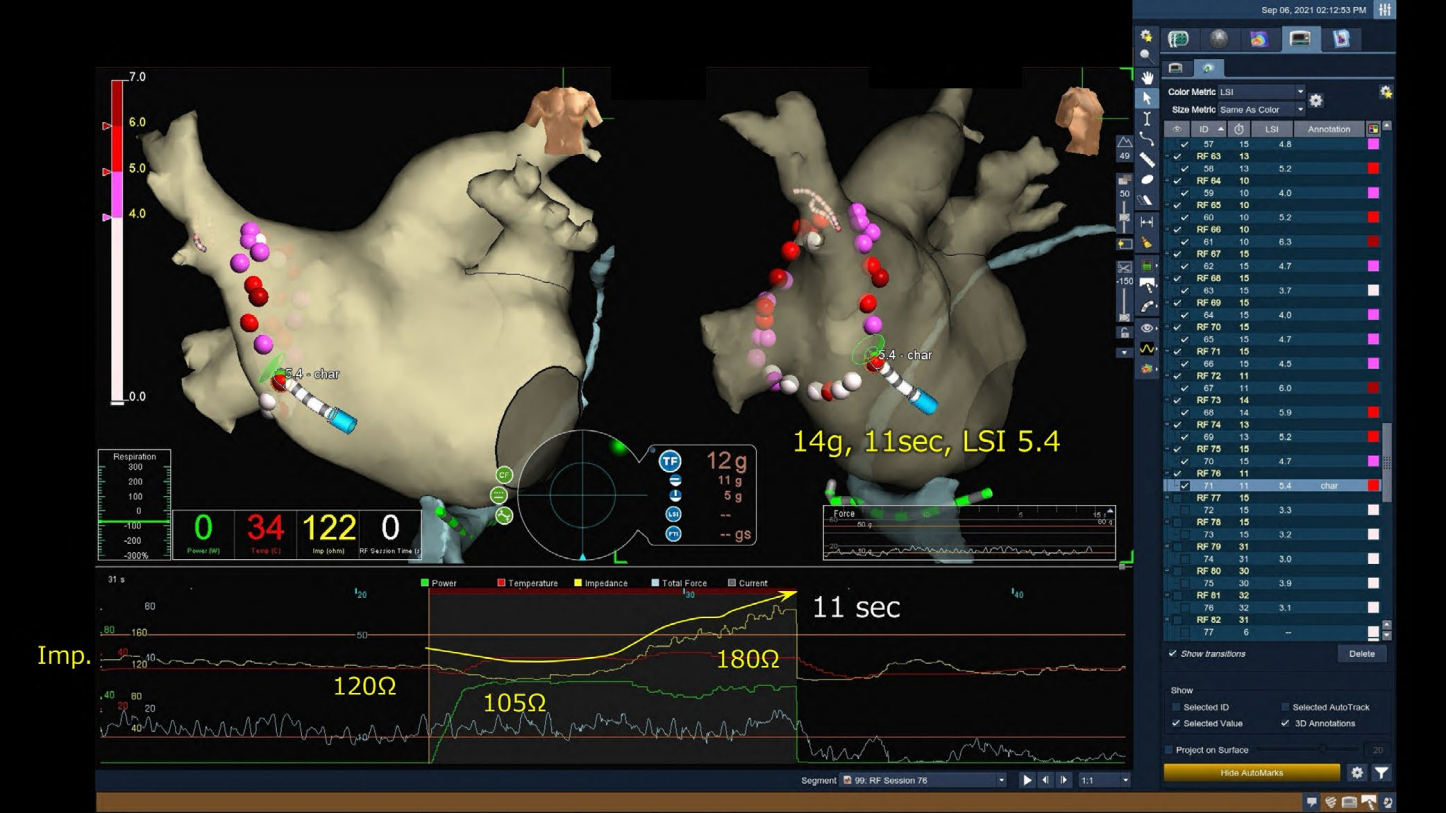
Impedance dynamics recorded during RF delivery at the site of the abrupt impedance rise. During circumferential ablation of the right pulmonary veins, high‐power, short‐duration ablation at the anterior wall of the right PVs, corresponding to the left atrial septal side, resulted in an abrupt impedance increase from 120 to 180 Ω, occurring 11 s after RF delivery initiation. Imp, impedance; LSI, Lesion size index.

**FIGURE 3:**
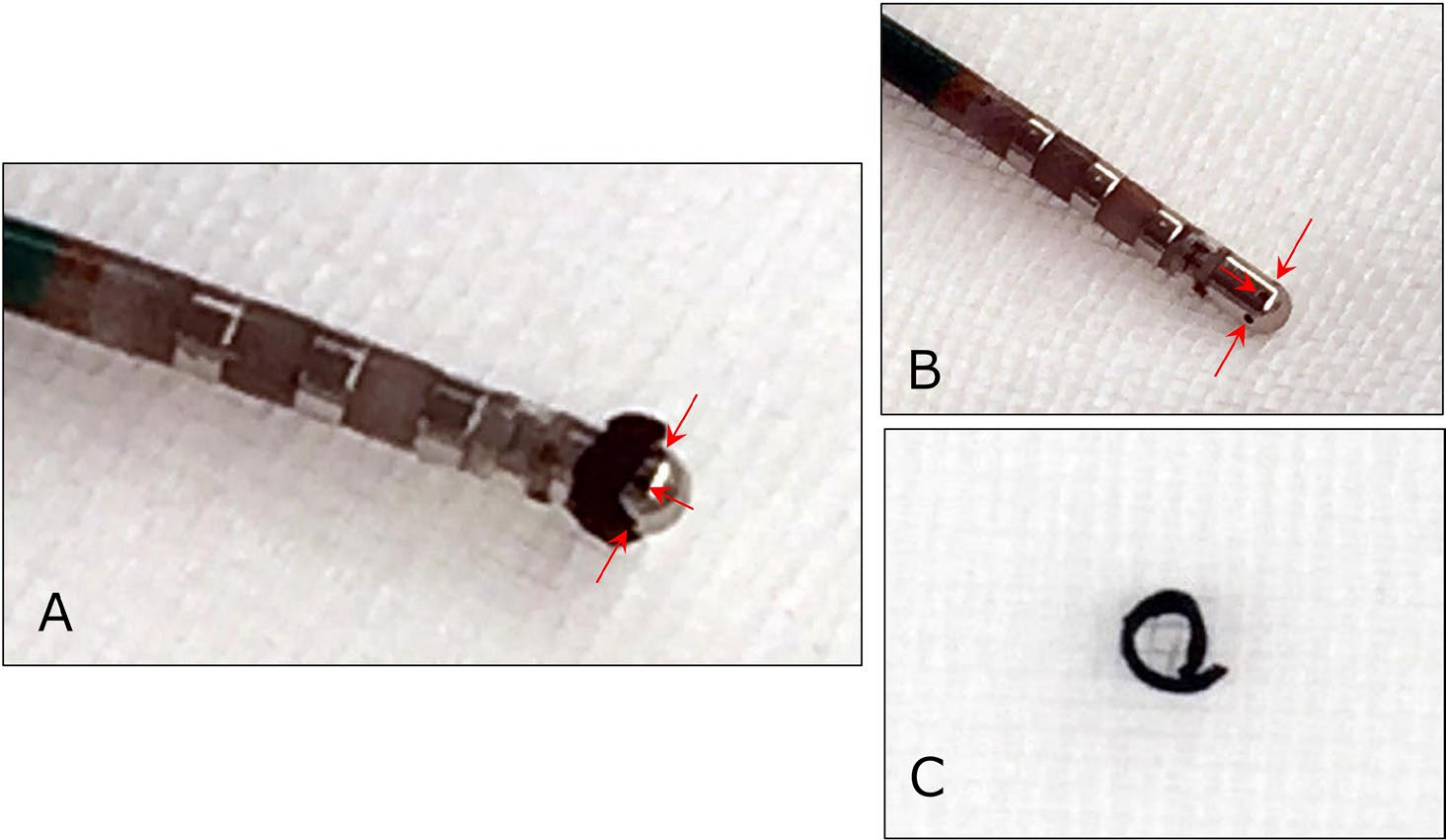
Ring char formation during extensive right pulmonary vein isolation. After an abrupt impedance rise, withdrawal of the ablation catheter from the long sheath revealed a circumferential thrombus adhering directly above the irrigation holes (red arrows) at the catheter tip (A). The catheter tip after thrombus removal is shown in (B), and the ring‐shaped thrombus that adhered to the catheter tip is displayed in (C).

A 70‐year‐old man with a history of cardioembolic stroke was referred for catheter ablation of paroxysmal AF. Echocardiography showed normal left ventricular function and a left atrial diameter of 37 mm. He had been receiving dabigatran 300 mg daily for one month, and the procedure was performed without interruption. After confirming the absence of left atrial thrombus on contrast‐enhanced CT, wide‐area circumferential PVI was conducted using the EnSite Velocity system (Abbott, MN, USA) and the TactiCath SE Ablation Catheter (Abbott). Ablation was performed with open irrigation and contact force sensing at 50 W with a temperature limit of 42°C. RF energy was applied for 10–15 s on the anterior wall, 10 s on the posterior wall, and 5–7 s near the esophagus, targeting a contact force of 5–10 g posteriorly and 5–15 g anteriorly (Figure 1). The lesion size index (LSI) target was 4.0–5.0, reduced to 3.5–4.0 near the esophagus. Esophageal temperature was continuously monitored using a probe with an integrated sensor, and energy delivery was halted if the temperature exceeded 40°C. Systemic heparinization maintained an activated clotting time of 350–400 s. PVI was performed on both sides during sinus rhythm. First pass isolation of the left PVs was achieved. During ablation of the right PVs, linear lesions were first created along the posterior wall, then the anterior wall. After 10 s of RF application at the anterior wall, the local impedance increased by 20 Ω, prompting termination. Subsequent ablation at the lower anterior wall of the right PVs, corresponding to the left atrial septal region, resulted in a decrease in local impedance from 120 to 105 Ω, followed by a rapid rise to 180 Ω (Figure 2). RF delivery was immediately terminated, and the application duration at this site was limited to 11 s. When the catheter was withdrawn, a ring‐shaped thrombus was found at its tip (Figure 3). The thrombus was removed, and ablation was resumed, achieving first pass isolation of the right PVs. No periprocedural embolic events occurred, and at 4‐year follow‐up, the patient remained free from AF recurrence.

## 3 Discussion

This case illustrates an unusual ring‐shaped char formation during HPSD ablation at the anterior wall of the right PVs. Although HPSD ablation shortens procedure time with low complication rates, char can still form under specific anatomic and technical conditions. Winkle et al. reported that using the TactiCath SE at 50 W for a mean duration of 11 s with 10–40 g contact force in 51 patients caused no char or stroke, confirming its safety [1]. However, our case suggests that char formation can still occur in vulnerable regions. Makimoto et al. found that excessive contact force (> 30 g) and long RF applications (> 20 s) were associated with gradual impedance rise and catheter tip charring, typically at the anteroinferior right PV [2]. Despite shorter application times in our case, local anatomic factors at the anterior right PV wall likely caused overheating and ring‐shaped char formation. Both our case and that reported by Makimoto et al. involved six‐hole irrigated catheters (TactiCath SE and THERMOCOOL SmartTouch [Biosense Webster, CA, USA], respectively). The case reported by Makimoto et al. also showed circumferential ring char just above the irrigation holes, similar to the findings observed in our case. This suggests a shared limitation of this design. Forceful contact against the septal myocardium can partially occlude irrigation holes, reducing cooling efficiency and predisposing the metallic surface above them to localized overheating. Furthermore, horizontal contact along the anterior right PV wall may cause the metallic tip to absorb resistive heat, producing proximal overheating, a mechanism described by other investigators [3]. In contrast, the newer **TactiFlex** catheter (Abbott), featuring a flexible tip with laser cut kerf irrigation, achieves more uniform cooling and reduces the risk of steam pops and thrombus formation [4]. Catheter design and procedural settings both influence char risk. Although the system used in this case was an older model that did not allow visualization of the catheter tip vector, even when using currently available vector‐displaying systems, a vector directed toward the septum during right anterior wall ablation represents a prerequisite. Excessive horizontal contact force may still result in occlusion of approximately half of the irrigation holes on one side of the catheter tip. Accordingly, careful attention is required when performing HPSD ablation, even with vector‐guided technology. As supporting evidence, Suga et al. found that using the QDOT MICRO catheter (Biosense Webster), which is equipped with 64 irrigation holes and designed to provide uniform cooling across the entire tip electrode, at 90 W for 4 s caused small but frequent char formation, particularly in the anterior inferior segment of the right inferior PV [5], indicating that even highly irrigated catheters may generate char under extreme power conditions.

To date, no clinical studies have directly evaluated the impact of char formation associated with high LSI values on procedural safety or long‐term clinical outcomes. Available evidence is largely limited to in vitro and preclinical studies, which indicate that higher LSI values, reflecting greater energy delivery and tissue heating, are mechanistically associated with an increased risk of tissue overheating and char formation. In an experimental study by Narita et al., ablation targeting a conventional LSI of 5.2 was directly compared with higher targets up to an LSI of 7.0 [6]. Although lesion size increased in a stable manner with higher LSI values, the incidence of steam pops was significantly higher at an LSI of 7.0, particularly under high‐power settings. Based on these findings, the authors cautioned against indiscriminately targeting high LSI values and emphasized the importance of integrating additional procedural parameters. In this context, although the LSI of 5.2 used in the present case is not generally considered excessive, our findings highlight that careful monitoring of impedance changes may provide critical safety information beyond the absolute LSI value alone.

The cooling effect and ablation profile of HPSD RF energy vary according to atrial rhythm. Hara et al. demonstrated that, despite similar ablation index values, which serve as ablation target metrics comparable to LSI, ablation during sinus rhythm results in more stable contact force and a greater impedance drop than ablation during AF, indicating rhythm‐dependent effects on lesion formation [7]. Although ablation in our case was performed during sinus rhythm, careful impedance monitoring remains essential because localized overheating and char formation can still occur under specific anatomic and contact conditions.

Clinically, char formation is significant because it can serve as a nidus for thrombus and embolization. Although large HPSD series have reported low complication rates, operators must remain alert. Careful monitoring of impedance, contact force, and catheter stability, especially in high‐risk regions like the anterior right PVs, is essential. Adjusting contact force, reducing power, or shortening applications at these sites may help prevent char formation. Impedance change remains an important safety indicator. Previous studies recommend stopping RF delivery immediately if impedance rises abruptly by ≥ 5–10 Ω from the nadir. In our case, energy delivery was terminated after an 80 Ω rise within 5 s, though a > 10 Ω increase had already occurred earlier. Earlier catheter withdrawal and inspection might have prevented thrombus formation, underscoring the importance of real‐time impedance monitoring and prompt catheter assessment to minimize thromboembolic risk.

Based on our experience with this case, we suggest avoiding HPSD ablation when using catheters equipped with only six irrigation holes. Furthermore, even when next‐generation catheters with improved irrigation and uniform tip cooling, such as the TactiFlex or QDOT MICRO, are used, HPSD ablation on the anterior wall of the right PV should be performed with careful catheter manipulation, avoiding excessive contact force and with continuous monitoring for any impedance rise).

## 4 Limitations

This report describes a single clinical case, which limits the generalizability of the findings. In addition, pathological or histological evaluation of the affected tissue was not performed, making definitive assessment of the underlying tissue changes impossible. Consequently, the exact mechanism cannot be determined with certainty. Although factors such as horizontal catheter tip contact, partial obstruction of irrigation holes, and local anatomical features may have contributed, their respective roles remain unclear.

## 5 Conclusions

Ring‐shaped char formation can occur even during HPSD ablation at the anterior wall of the right PVs. Although this technique is generally safe and efficient, anatomical constraints and impaired irrigation may predispose to localized overheating. Continuous monitoring of impedance, contact force, and lesion indices, together with consideration of catheter design, is crucial to minimize thromboembolic risk and ensure procedural safety.

## Funding

This case report did not receive any specific grants from funding agencies in the public, commercial, or not‐for‐profit sectors.

## Ethics Statement

This study was approved by the Yokohama City Bay Hospital Ethics Committee.

## Consent

Written informed consent was obtained from the patient.

## Conflicts of Interest

The authors declare no conflicts of interest.

## Data Availability

The data that support the findings of this study are available from the corresponding author upon reasonable request.
